# (*S*)-(−)-5,5′-Bis(diphenyl­phosphino)-4,4′-bi-1,3-benzodioxole

**DOI:** 10.1107/S1600536809015335

**Published:** 2009-04-30

**Authors:** Ling-Yan Jian, Xiao-Jing He, Ya-Xin Sun, Qing-Hua Jiang, Xu Zhu

**Affiliations:** a Shengjing Hospital of China Medical University, Shenyang 110004, People’s Republic of China

## Abstract

In the chiral title compound, C_38_H_28_O_4_P_2_, the intra­molecular P⋯P separation is 3.671 (2) Å and the dihedral angle between the two benzene rings in the biphenyl unit is 77.9 (2)°.

## Related literature

For background on asymmetric synthesis and catalysis using this type of chiral ligand, see: Horner *et al.* (1968 ▶); Aikawa *et al.* (2004 ▶). For the synthesis, see: Saito *et al.* (2001 ▶). For a related structure, see: Jones *et al.* (2003 ▶).
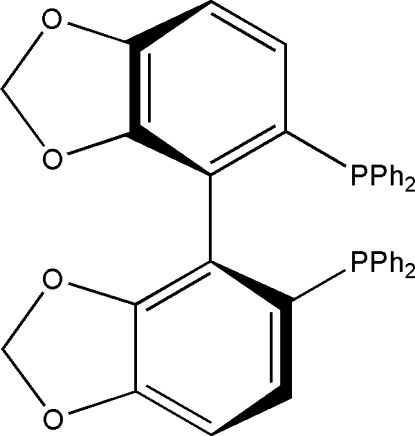


## Experimental

### 

#### Crystal data

C_38_H_28_O_4_P_2_
*M* *_r_* = 610.54
Orthorhombic, 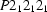

*a* = 10.4735 (10) Å
*b* = 15.8362 (15) Å
*c* = 18.7349 (17) Å
*V* = 3107.4 (5) Å^3^
*Z* = 4
Mo *K*α radiation
μ = 0.18 mm^−1^
*T* = 295 K
0.22 × 0.20 × 0.17 mm

#### Data collection

Siemens SMART CCD diffractometer
Absorption correction: multi-scan (*SADABS*; Siemens, 1996 ▶) *T*_min_ = 0.961, *T*_max_ = 0.970
16522 measured reflections
5515 independent reflections
4665 reflections with *I* > 2σ(*I*)
*R*_int_ = 0.031

#### Refinement

*R*[*F* ^2^ > 2σ(*F* ^2^)] = 0.034
*wR*(*F* ^2^) = 0.083
*S* = 1.03
5515 reflections
397 parameters
H-atom parameters constrained
Δρ_max_ = 0.25 e Å^−3^
Δρ_min_ = −0.16 e Å^−3^
Absolute structure: Flack (1983 ▶), 2403 Friedel pairs
Flack parameter: 0.06 (8)

### 

Data collection: *SMART* (Siemens, 1996 ▶); cell refinement: *SAINT* (Siemens, 1996 ▶); data reduction: *SAINT*; program(s) used to solve structure: *SHELXS97* (Sheldrick, 2008 ▶); program(s) used to refine structure: *SHELXL97* (Sheldrick, 2008 ▶); molecular graphics: *SHELXTL* (Sheldrick, 2008 ▶); software used to prepare material for publication: *SHELXTL*.

## Supplementary Material

Crystal structure: contains datablocks global, I. DOI: 10.1107/S1600536809015335/hb2954sup1.cif

Structure factors: contains datablocks I. DOI: 10.1107/S1600536809015335/hb2954Isup2.hkl

Additional supplementary materials:  crystallographic information; 3D view; checkCIF report

## Figures and Tables

**Fig. 1.**
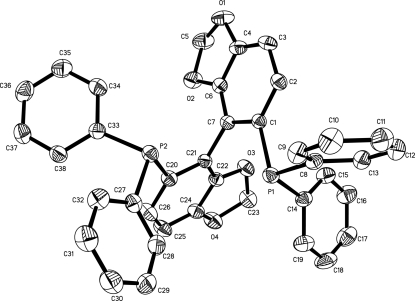
The molecular structure of (I), with displacement ellipsoids drawn at the 30% probability level. H atoms have been omitted for clarity.

## supplementary crystallographic information

### Comment

The ability to selectively form one enantiomer in preference to the other (asymmetric catalysis) is undoubtedly one of the major advances in modern drug design and synthesis. Since 1968, when a chiral phosphine was first utilized in asymmetric hydrogenation (Horner *et al.*, 1968), much effort has been devoted to the design and synthesis of chiral phosphine ligands. The synthesis of the title compound has been reported in literature (Saito *et al.*, 2001). However, this is the first time that the crystal structure is being reported. This ligand has been used on palladium for the catalysis of ketone-ene reactions (Aikawa *et al.*, 2004). All bond lengths and angles in (I) are normal and are comparable to those in the related compound (*S*)-(–)-2,2'-Bis(diphenylphosphino) -1,1'-binaphthyl (Jones *et al.*, 2003). The key feature of (I) is the intramolecular P···P distance of 3.671 (2) Å and the two benzene rings in the biphenyl moiety make a dihedral angle of 77.9 (2)°.

### Experimental

The title compound was synthesized by the literature route of Saito *et al.* (2001). Colourless blocks of (I) were grown by slow evaporation of a solution of the compound in a acetone-ethanol (1:1 v/v) mixture.

### Refinement

All the H atoms were initially located in a difference map, relocated in idealised positions (C—H = 0.93–0.97 Å) and refined as riding with *U*_iso_(H) = 1.2*U*_eq_(C).

### Crystal data

**Table:**

C_38_H_28_O_4_P_2_	*F*(000) = 1272
*M**_r_* = 610.54	*D*_x_ = 1.305 Mg m^−^^3^
Orthorhombic, *P*2_1_2_1_2_1_	Mo *K*α radiation, λ = 0.71073 Å
Hall symbol: P 2ac 2ab	Cell parameters from 4758 reflections
*a* = 10.4735 (10) Å	θ = 2.2–23.5°
*b* = 15.8362 (15) Å	µ = 0.18 mm^−^^1^
*c* = 18.7349 (17) Å	*T* = 295 K
*V* = 3107.4 (5) Å^3^	Block, colourless
*Z* = 4	0.22 × 0.20 × 0.17 mm

### Data collection

**Table:**

Siemens SMART CCD diffractometer	5515 independent reflections
Radiation source: fine-focus sealed tube	4665 reflections with *I* > 2σ(*I*)
graphite	*R*_int_ = 0.031
ω scans	θ_max_ = 25.1°, θ_min_ = 1.7°
Absorption correction: multi-scan (*SADABS*; Siemens, 1996)	*h* = −12→8
*T*_min_ = 0.961, *T*_max_ = 0.970	*k* = −18→18
16522 measured reflections	*l* = −20→22

### Refinement

**Table:**

Refinement on *F*^2^	Secondary atom site location: difference Fourier map
Least-squares matrix: full	Hydrogen site location: inferred from neighbouring sites
*R*[*F*^2^ > 2σ(*F*^2^)] = 0.034	H-atom parameters constrained
*wR*(*F*^2^) = 0.083	*w* = 1/[σ^2^(*F*_o_^2^) + (0.0405*P*)^2^ + 0.2496*P*] where *P* = (*F*_o_^2^ + 2*F*_c_^2^)/3
*S* = 1.03	(Δ/σ)_max_ = 0.001
5515 reflections	Δρ_max_ = 0.25 e Å^−^^3^
397 parameters	Δρ_min_ = −0.16 e Å^−^^3^
0 restraints	Absolute structure: Flack (1983), 2403 Friedel pairs
Primary atom site location: structure-invariant direct methods	Flack parameter: 0.06 (8)

### Special details

**Table:**

Geometry. All e.s.d.'s (except the e.s.d. in the dihedral angle between two l.s. planes) are estimated using the full covariance matrix. The cell e.s.d.'s are taken into account individually in the estimation of e.s.d.'s in distances, angles and torsion angles; correlations between e.s.d.'s in cell parameters are only used when they are defined by crystal symmetry. An approximate (isotropic) treatment of cell e.s.d.'s is used for estimating e.s.d.'s involving l.s. planes.
Refinement. Refinement of *F*^2^ against ALL reflections. The weighted *R*-factor *wR* and goodness of fit *S* are based on *F*^2^, conventional *R*-factors *R* are based on *F*, with *F* set to zero for negative *F*^2^. The threshold expression of *F*^2^ > σ(*F*^2^) is used only for calculating *R*-factors(gt) *etc*. and is not relevant to the choice of reflections for refinement. *R*-factors based on *F*^2^ are statistically about twice as large as those based on *F*, and *R*- factors based on ALL data will be even larger.

### Fractional atomic coordinates and isotropic or equivalent isotropic displacement parameters (Å^2^)

**Table:**

	*x*	*y*	*z*	*U*_iso_*/*U*_eq_	
P1	0.58912 (6)	0.58094 (4)	0.84832 (3)	0.04188 (15)	
P2	0.59298 (6)	0.53283 (4)	0.65667 (3)	0.04263 (15)	
O1	0.13104 (16)	0.70954 (11)	0.69648 (11)	0.0662 (5)	
O2	0.33097 (16)	0.74812 (10)	0.65651 (9)	0.0569 (4)	
O3	0.57161 (16)	0.83894 (9)	0.76483 (9)	0.0518 (4)	
O4	0.76116 (17)	0.88509 (10)	0.71511 (10)	0.0597 (5)	
C1	0.4390 (2)	0.61654 (13)	0.80753 (11)	0.0355 (5)	
C2	0.3188 (2)	0.59414 (14)	0.83092 (12)	0.0432 (5)	
H2	0.3117	0.5596	0.8709	0.052*	
C3	0.2068 (2)	0.62152 (15)	0.79677 (14)	0.0499 (6)	
H3	0.1262	0.6048	0.8122	0.060*	
C4	0.2228 (2)	0.67396 (15)	0.73985 (13)	0.0461 (6)	
C5	0.1990 (3)	0.77173 (19)	0.65656 (17)	0.0694 (8)	
H5A	0.1885	0.8269	0.6783	0.083*	
H5B	0.1668	0.7742	0.6081	0.083*	
C6	0.3422 (2)	0.69712 (13)	0.71610 (12)	0.0402 (5)	
C7	0.4530 (2)	0.66937 (13)	0.74655 (11)	0.0359 (5)	
C8	0.5330 (2)	0.51007 (14)	0.91959 (11)	0.0406 (5)	
C9	0.5239 (3)	0.42500 (15)	0.90265 (13)	0.0541 (6)	
H9	0.5446	0.4071	0.8568	0.065*	
C10	0.4845 (3)	0.36647 (17)	0.95267 (16)	0.0665 (8)	
H10	0.4774	0.3098	0.9404	0.080*	
C11	0.4561 (3)	0.39259 (18)	1.02055 (15)	0.0652 (8)	
H11	0.4300	0.3534	1.0545	0.078*	
C12	0.4660 (2)	0.47679 (18)	1.03892 (13)	0.0589 (7)	
H12	0.4472	0.4942	1.0852	0.071*	
C13	0.5039 (2)	0.53493 (16)	0.98843 (12)	0.0478 (6)	
H13	0.5099	0.5916	1.0008	0.057*	
C14	0.6373 (2)	0.67684 (15)	0.89654 (12)	0.0440 (6)	
C15	0.5534 (3)	0.73889 (16)	0.91921 (13)	0.0544 (7)	
H15	0.4665	0.7325	0.9107	0.065*	
C16	0.5973 (3)	0.81080 (17)	0.95458 (15)	0.0649 (7)	
H16	0.5395	0.8516	0.9699	0.078*	
C17	0.7256 (3)	0.82148 (19)	0.96687 (16)	0.0703 (8)	
H17	0.7552	0.8691	0.9907	0.084*	
C18	0.8092 (3)	0.7612 (2)	0.94363 (17)	0.0736 (9)	
H18	0.8962	0.7686	0.9514	0.088*	
C19	0.7667 (3)	0.68914 (17)	0.90862 (14)	0.0608 (7)	
H19	0.8252	0.6489	0.8932	0.073*	
C20	0.6520 (2)	0.64110 (13)	0.67129 (12)	0.0419 (5)	
C21	0.5790 (2)	0.69450 (13)	0.71579 (11)	0.0360 (5)	
C22	0.6264 (2)	0.77484 (14)	0.72645 (12)	0.0400 (5)	
C23	0.6690 (3)	0.90246 (15)	0.76970 (14)	0.0581 (7)	
H23A	0.6323	0.9581	0.7628	0.070*	
H23B	0.7091	0.9008	0.8163	0.070*	
C24	0.7386 (2)	0.80219 (14)	0.69604 (13)	0.0463 (6)	
C25	0.8098 (2)	0.75234 (15)	0.65207 (15)	0.0568 (7)	
H25	0.8847	0.7716	0.6309	0.068*	
C26	0.7641 (2)	0.67079 (15)	0.64068 (14)	0.0538 (6)	
H26	0.8106	0.6347	0.6114	0.065*	
C27	0.7379 (2)	0.46870 (14)	0.66098 (12)	0.0443 (5)	
C28	0.8398 (3)	0.48831 (17)	0.70534 (13)	0.0580 (7)	
H28	0.8393	0.5391	0.7303	0.070*	
C29	0.9414 (3)	0.4341 (2)	0.71318 (14)	0.0687 (8)	
H29	1.0092	0.4491	0.7427	0.082*	
C30	0.9438 (3)	0.3586 (2)	0.67796 (15)	0.0732 (9)	
H30	1.0125	0.3220	0.6838	0.088*	
C31	0.8450 (3)	0.33712 (17)	0.63417 (16)	0.0689 (8)	
H31	0.8466	0.2859	0.6099	0.083*	
C32	0.7425 (3)	0.39115 (15)	0.62571 (14)	0.0580 (7)	
H32	0.6754	0.3755	0.5960	0.070*	
C33	0.5558 (2)	0.53725 (14)	0.56069 (12)	0.0433 (5)	
C34	0.4283 (2)	0.55116 (16)	0.54330 (14)	0.0575 (7)	
H34	0.3679	0.5560	0.5795	0.069*	
C35	0.3904 (3)	0.55778 (19)	0.47284 (15)	0.0703 (8)	
H35	0.3053	0.5683	0.4619	0.084*	
C36	0.4780 (3)	0.54888 (18)	0.41902 (14)	0.0660 (8)	
H36	0.4523	0.5530	0.3716	0.079*	
C37	0.6031 (3)	0.53395 (17)	0.43509 (13)	0.0596 (7)	
H37	0.6621	0.5272	0.3984	0.072*	
C38	0.6431 (2)	0.52869 (16)	0.50546 (12)	0.0526 (6)	
H38	0.7289	0.5194	0.5157	0.063*	

### Atomic displacement parameters (Å^2^)

**Table:**

	*U*^11^	*U*^22^	*U*^33^	*U*^12^	*U*^13^	*U*^23^
P1	0.0382 (3)	0.0438 (3)	0.0437 (3)	0.0045 (3)	0.0006 (3)	0.0023 (3)
P2	0.0426 (3)	0.0394 (3)	0.0459 (3)	−0.0018 (3)	0.0061 (3)	−0.0014 (3)
O1	0.0412 (10)	0.0677 (12)	0.0899 (14)	0.0021 (9)	−0.0192 (9)	0.0103 (11)
O2	0.0532 (10)	0.0582 (11)	0.0592 (10)	0.0064 (8)	−0.0073 (9)	0.0185 (9)
O3	0.0507 (10)	0.0390 (9)	0.0656 (11)	−0.0013 (8)	0.0148 (8)	−0.0060 (8)
O4	0.0539 (11)	0.0432 (10)	0.0821 (12)	−0.0109 (8)	0.0146 (10)	−0.0049 (9)
C1	0.0357 (12)	0.0337 (11)	0.0373 (11)	0.0005 (9)	0.0019 (9)	−0.0017 (9)
C2	0.0429 (13)	0.0415 (13)	0.0450 (13)	−0.0033 (10)	0.0062 (11)	0.0020 (11)
C3	0.0339 (13)	0.0518 (15)	0.0640 (16)	−0.0072 (11)	0.0047 (12)	0.0000 (13)
C4	0.0353 (13)	0.0432 (13)	0.0599 (15)	0.0029 (11)	−0.0070 (11)	−0.0050 (12)
C5	0.0551 (17)	0.0711 (18)	0.082 (2)	0.0082 (14)	−0.0180 (16)	0.0153 (17)
C6	0.0420 (13)	0.0360 (12)	0.0427 (12)	0.0032 (10)	−0.0004 (11)	−0.0010 (10)
C7	0.0352 (11)	0.0319 (11)	0.0406 (11)	−0.0014 (10)	0.0023 (10)	−0.0035 (9)
C8	0.0400 (13)	0.0379 (13)	0.0438 (12)	0.0033 (10)	−0.0059 (10)	0.0017 (10)
C9	0.0653 (17)	0.0457 (14)	0.0514 (14)	0.0057 (13)	−0.0047 (13)	−0.0014 (12)
C10	0.087 (2)	0.0408 (14)	0.0721 (19)	−0.0049 (14)	−0.0048 (17)	0.0085 (14)
C11	0.0703 (19)	0.0642 (18)	0.0612 (17)	−0.0080 (14)	−0.0057 (15)	0.0241 (15)
C12	0.0607 (17)	0.0720 (19)	0.0439 (14)	−0.0015 (14)	−0.0014 (12)	0.0045 (14)
C13	0.0515 (14)	0.0477 (13)	0.0442 (13)	0.0031 (12)	−0.0024 (11)	−0.0005 (12)
C14	0.0413 (13)	0.0462 (13)	0.0444 (13)	−0.0037 (11)	−0.0049 (10)	0.0084 (11)
C15	0.0477 (16)	0.0526 (15)	0.0631 (16)	−0.0027 (12)	−0.0066 (12)	−0.0030 (13)
C16	0.0681 (19)	0.0528 (15)	0.0737 (18)	−0.0040 (15)	−0.0105 (16)	−0.0095 (14)
C17	0.080 (2)	0.0600 (17)	0.0712 (19)	−0.0237 (17)	−0.0169 (17)	−0.0010 (15)
C18	0.0518 (18)	0.086 (2)	0.083 (2)	−0.0229 (18)	−0.0204 (16)	0.0056 (18)
C19	0.0469 (15)	0.0660 (18)	0.0694 (18)	−0.0028 (13)	−0.0099 (14)	0.0054 (15)
C20	0.0418 (13)	0.0382 (12)	0.0459 (13)	−0.0038 (10)	0.0028 (11)	0.0011 (10)
C21	0.0336 (11)	0.0405 (12)	0.0339 (11)	0.0006 (10)	0.0012 (10)	0.0059 (9)
C22	0.0368 (13)	0.0385 (12)	0.0447 (13)	0.0018 (10)	0.0017 (10)	0.0042 (11)
C23	0.0644 (17)	0.0405 (14)	0.0695 (17)	−0.0057 (13)	0.0094 (14)	−0.0018 (13)
C24	0.0414 (13)	0.0397 (13)	0.0577 (15)	−0.0038 (11)	0.0036 (12)	0.0022 (11)
C25	0.0441 (14)	0.0530 (16)	0.0734 (17)	−0.0072 (12)	0.0185 (14)	−0.0015 (14)
C26	0.0488 (14)	0.0477 (14)	0.0648 (16)	−0.0005 (12)	0.0209 (13)	−0.0060 (13)
C27	0.0494 (13)	0.0410 (12)	0.0427 (12)	0.0027 (11)	0.0052 (11)	0.0049 (11)
C28	0.0612 (17)	0.0672 (17)	0.0457 (14)	0.0074 (14)	−0.0004 (13)	−0.0052 (13)
C29	0.0667 (19)	0.091 (2)	0.0487 (15)	0.0180 (17)	−0.0089 (14)	−0.0006 (15)
C30	0.074 (2)	0.083 (2)	0.0626 (18)	0.0319 (17)	0.0057 (16)	0.0224 (16)
C31	0.085 (2)	0.0463 (15)	0.075 (2)	0.0162 (15)	0.0055 (17)	0.0044 (14)
C32	0.0641 (17)	0.0459 (15)	0.0639 (16)	0.0037 (13)	−0.0066 (14)	0.0035 (13)
C33	0.0415 (14)	0.0377 (12)	0.0507 (13)	0.0012 (10)	0.0002 (11)	−0.0044 (11)
C34	0.0472 (15)	0.0657 (17)	0.0596 (15)	0.0099 (13)	0.0031 (13)	−0.0031 (13)
C35	0.0551 (17)	0.085 (2)	0.0706 (19)	0.0149 (15)	−0.0136 (15)	−0.0028 (15)
C36	0.079 (2)	0.0689 (19)	0.0498 (15)	0.0104 (16)	−0.0144 (15)	0.0018 (13)
C37	0.0664 (19)	0.0635 (16)	0.0489 (14)	0.0081 (16)	0.0090 (13)	0.0026 (13)
C38	0.0444 (14)	0.0623 (15)	0.0511 (14)	0.0081 (13)	0.0030 (11)	0.0020 (13)

### Geometric parameters (Å, °)

**Table:**

P1—C1	1.837 (2)	C16—C17	1.374 (4)
P1—C14	1.838 (2)	C16—H16	0.9300
P1—C8	1.841 (2)	C17—C18	1.367 (4)
P2—C27	1.828 (2)	C17—H17	0.9300
P2—C33	1.841 (2)	C18—C19	1.390 (4)
P2—C20	1.843 (2)	C18—H18	0.9300
O1—C4	1.379 (3)	C19—H19	0.9300
O1—C5	1.427 (3)	C20—C26	1.389 (3)
O2—C6	1.383 (3)	C20—C21	1.412 (3)
O2—C5	1.432 (3)	C21—C22	1.380 (3)
O3—C22	1.370 (3)	C22—C24	1.375 (3)
O3—C23	1.435 (3)	C23—H23A	0.9700
O4—C24	1.381 (3)	C23—H23B	0.9700
O4—C23	1.433 (3)	C24—C25	1.363 (3)
C1—C2	1.379 (3)	C25—C26	1.394 (3)
C1—C7	1.424 (3)	C25—H25	0.9300
C2—C3	1.405 (3)	C26—H26	0.9300
C2—H2	0.9300	C27—C28	1.388 (3)
C3—C4	1.362 (3)	C27—C32	1.395 (3)
C3—H3	0.9300	C28—C29	1.375 (4)
C4—C6	1.377 (3)	C28—H28	0.9300
C5—H5A	0.9700	C29—C30	1.367 (4)
C5—H5B	0.9700	C29—H29	0.9300
C6—C7	1.366 (3)	C30—C31	1.364 (4)
C7—C21	1.494 (3)	C30—H30	0.9300
C8—C13	1.382 (3)	C31—C32	1.382 (4)
C8—C9	1.387 (3)	C31—H31	0.9300
C9—C10	1.381 (4)	C32—H32	0.9300
C9—H9	0.9300	C33—C38	1.388 (3)
C10—C11	1.370 (4)	C33—C34	1.392 (3)
C10—H10	0.9300	C34—C35	1.382 (4)
C11—C12	1.381 (4)	C34—H34	0.9300
C11—H11	0.9300	C35—C36	1.371 (4)
C12—C13	1.378 (3)	C35—H35	0.9300
C12—H12	0.9300	C36—C37	1.365 (4)
C13—H13	0.9300	C36—H36	0.9300
C14—C15	1.385 (3)	C37—C38	1.386 (3)
C14—C19	1.388 (3)	C37—H37	0.9300
C15—C16	1.395 (3)	C38—H38	0.9300
C15—H15	0.9300		
			
C1—P1—C14	100.70 (10)	C17—C18—H18	119.4
C1—P1—C8	102.44 (10)	C19—C18—H18	119.4
C14—P1—C8	103.58 (10)	C14—C19—C18	120.4 (3)
C27—P2—C33	103.89 (10)	C14—C19—H19	119.8
C27—P2—C20	103.41 (11)	C18—C19—H19	119.8
C33—P2—C20	100.41 (10)	C26—C20—C21	119.9 (2)
C4—O1—C5	104.07 (19)	C26—C20—P2	122.49 (18)
C6—O2—C5	103.55 (19)	C21—C20—P2	117.62 (16)
C22—O3—C23	104.76 (18)	C22—C21—C20	116.30 (19)
C24—O4—C23	104.59 (18)	C22—C21—C7	120.49 (19)
C2—C1—C7	120.04 (19)	C20—C21—C7	123.10 (19)
C2—C1—P1	124.75 (16)	O3—C22—C24	109.99 (19)
C7—C1—P1	115.19 (15)	O3—C22—C21	127.47 (19)
C1—C2—C3	122.6 (2)	C24—C22—C21	122.5 (2)
C1—C2—H2	118.7	O4—C23—O3	107.39 (18)
C3—C2—H2	118.7	O4—C23—H23A	110.2
C4—C3—C2	116.2 (2)	O3—C23—H23A	110.2
C4—C3—H3	121.9	O4—C23—H23B	110.2
C2—C3—H3	121.9	O3—C23—H23B	110.2
C3—C4—C6	121.8 (2)	H23A—C23—H23B	108.5
C3—C4—O1	128.6 (2)	C25—C24—C22	122.4 (2)
C6—C4—O1	109.5 (2)	C25—C24—O4	127.8 (2)
O1—C5—O2	107.6 (2)	C22—C24—O4	109.8 (2)
O1—C5—H5A	110.2	C24—C25—C26	116.2 (2)
O2—C5—H5A	110.2	C24—C25—H25	121.9
O1—C5—H5B	110.2	C26—C25—H25	121.9
O2—C5—H5B	110.2	C20—C26—C25	122.8 (2)
H5A—C5—H5B	108.5	C20—C26—H26	118.6
C7—C6—C4	123.4 (2)	C25—C26—H26	118.6
C7—C6—O2	126.7 (2)	C28—C27—C32	117.0 (2)
C4—C6—O2	109.8 (2)	C28—C27—P2	122.72 (18)
C6—C7—C1	115.87 (19)	C32—C27—P2	119.78 (19)
C6—C7—C21	120.25 (19)	C29—C28—C27	121.3 (2)
C1—C7—C21	123.88 (18)	C29—C28—H28	119.3
C13—C8—C9	118.3 (2)	C27—C28—H28	119.3
C13—C8—P1	125.00 (17)	C30—C29—C28	120.6 (3)
C9—C8—P1	116.62 (18)	C30—C29—H29	119.7
C10—C9—C8	121.1 (2)	C28—C29—H29	119.7
C10—C9—H9	119.4	C31—C30—C29	119.6 (3)
C8—C9—H9	119.4	C31—C30—H30	120.2
C11—C10—C9	119.5 (3)	C29—C30—H30	120.2
C11—C10—H10	120.3	C30—C31—C32	120.3 (3)
C9—C10—H10	120.3	C30—C31—H31	119.9
C10—C11—C12	120.4 (2)	C32—C31—H31	119.9
C10—C11—H11	119.8	C31—C32—C27	121.2 (3)
C12—C11—H11	119.8	C31—C32—H32	119.4
C13—C12—C11	119.7 (2)	C27—C32—H32	119.4
C13—C12—H12	120.1	C38—C33—C34	118.3 (2)
C11—C12—H12	120.1	C38—C33—P2	125.78 (18)
C12—C13—C8	120.9 (2)	C34—C33—P2	115.96 (18)
C12—C13—H13	119.6	C35—C34—C33	120.7 (2)
C8—C13—H13	119.6	C35—C34—H34	119.6
C15—C14—C19	118.0 (2)	C33—C34—H34	119.6
C15—C14—P1	124.29 (18)	C36—C35—C34	120.2 (3)
C19—C14—P1	117.7 (2)	C36—C35—H35	119.9
C14—C15—C16	121.1 (3)	C34—C35—H35	119.9
C14—C15—H15	119.5	C37—C36—C35	119.9 (3)
C16—C15—H15	119.5	C37—C36—H36	120.1
C17—C16—C15	120.1 (3)	C35—C36—H36	120.1
C17—C16—H16	119.9	C36—C37—C38	120.7 (3)
C15—C16—H16	119.9	C36—C37—H37	119.7
C18—C17—C16	119.2 (3)	C38—C37—H37	119.7
C18—C17—H17	120.4	C37—C38—C33	120.3 (2)
C16—C17—H17	120.4	C37—C38—H38	119.9
C17—C18—C19	121.2 (3)	C33—C38—H38	119.9
			
C14—P1—C1—C2	−104.4 (2)	C27—P2—C20—C21	−138.78 (17)
C8—P1—C1—C2	2.2 (2)	C33—P2—C20—C21	114.08 (18)
C14—P1—C1—C7	77.10 (17)	C26—C20—C21—C22	−0.4 (3)
C8—P1—C1—C7	−176.25 (15)	P2—C20—C21—C22	179.22 (16)
C7—C1—C2—C3	0.0 (3)	C26—C20—C21—C7	175.6 (2)
P1—C1—C2—C3	−178.44 (18)	P2—C20—C21—C7	−4.7 (3)
C1—C2—C3—C4	−1.8 (3)	C6—C7—C21—C22	75.5 (3)
C2—C3—C4—C6	1.5 (4)	C1—C7—C21—C22	−104.6 (2)
C2—C3—C4—O1	179.4 (2)	C6—C7—C21—C20	−100.4 (3)
C5—O1—C4—C3	168.0 (3)	C1—C7—C21—C20	79.5 (3)
C5—O1—C4—C6	−14.0 (3)	C23—O3—C22—C24	−12.2 (3)
C4—O1—C5—O2	22.8 (3)	C23—O3—C22—C21	169.9 (2)
C6—O2—C5—O1	−22.8 (3)	C20—C21—C22—O3	177.4 (2)
C3—C4—C6—C7	0.7 (4)	C7—C21—C22—O3	1.2 (3)
O1—C4—C6—C7	−177.6 (2)	C20—C21—C22—C24	−0.3 (3)
C3—C4—C6—O2	178.0 (2)	C7—C21—C22—C24	−176.4 (2)
O1—C4—C6—O2	−0.2 (3)	C24—O4—C23—O3	−17.8 (3)
C5—O2—C6—C7	−168.5 (2)	C22—O3—C23—O4	18.5 (3)
C5—O2—C6—C4	14.2 (3)	O3—C22—C24—C25	−176.8 (2)
C4—C6—C7—C1	−2.5 (3)	C21—C22—C24—C25	1.2 (4)
O2—C6—C7—C1	−179.40 (19)	O3—C22—C24—O4	1.1 (3)
C4—C6—C7—C21	177.4 (2)	C21—C22—C24—O4	179.2 (2)
O2—C6—C7—C21	0.5 (3)	C23—O4—C24—C25	−171.8 (3)
C2—C1—C7—C6	2.1 (3)	C23—O4—C24—C22	10.4 (3)
P1—C1—C7—C6	−179.30 (16)	C22—C24—C25—C26	−1.4 (4)
C2—C1—C7—C21	−177.8 (2)	O4—C24—C25—C26	−178.9 (2)
P1—C1—C7—C21	0.8 (3)	C21—C20—C26—C25	0.3 (4)
C1—P1—C8—C13	−89.2 (2)	P2—C20—C26—C25	−179.4 (2)
C14—P1—C8—C13	15.2 (2)	C24—C25—C26—C20	0.6 (4)
C1—P1—C8—C9	93.1 (2)	C33—P2—C27—C28	138.3 (2)
C14—P1—C8—C9	−162.46 (19)	C20—P2—C27—C28	33.8 (2)
C13—C8—C9—C10	1.2 (4)	C33—P2—C27—C32	−50.1 (2)
P1—C8—C9—C10	179.0 (2)	C20—P2—C27—C32	−154.56 (19)
C8—C9—C10—C11	−1.1 (4)	C32—C27—C28—C29	1.0 (4)
C9—C10—C11—C12	0.3 (4)	P2—C27—C28—C29	172.9 (2)
C10—C11—C12—C13	0.5 (4)	C27—C28—C29—C30	−0.9 (4)
C11—C12—C13—C8	−0.4 (4)	C28—C29—C30—C31	0.6 (4)
C9—C8—C13—C12	−0.4 (4)	C29—C30—C31—C32	−0.4 (4)
P1—C8—C13—C12	−178.04 (19)	C30—C31—C32—C27	0.5 (4)
C1—P1—C14—C15	24.7 (2)	C28—C27—C32—C31	−0.8 (4)
C8—P1—C14—C15	−81.1 (2)	P2—C27—C32—C31	−172.9 (2)
C1—P1—C14—C19	−152.86 (19)	C27—P2—C33—C38	−25.8 (2)
C8—P1—C14—C19	101.4 (2)	C20—P2—C33—C38	81.0 (2)
C19—C14—C15—C16	−1.4 (4)	C27—P2—C33—C34	154.78 (19)
P1—C14—C15—C16	−178.93 (19)	C20—P2—C33—C34	−98.5 (2)
C14—C15—C16—C17	0.7 (4)	C38—C33—C34—C35	−1.2 (4)
C15—C16—C17—C18	0.4 (4)	P2—C33—C34—C35	178.3 (2)
C16—C17—C18—C19	−0.7 (5)	C33—C34—C35—C36	1.4 (4)
C15—C14—C19—C18	1.1 (4)	C34—C35—C36—C37	−0.4 (5)
P1—C14—C19—C18	178.8 (2)	C35—C36—C37—C38	−0.8 (5)
C17—C18—C19—C14	−0.1 (4)	C36—C37—C38—C33	1.0 (4)
C27—P2—C20—C26	40.9 (2)	C34—C33—C38—C37	0.0 (4)
C33—P2—C20—C26	−66.3 (2)	P2—C33—C38—C37	−179.5 (2)
